# Supplementary material for: Genome-scale CRISPR screens identify host factors that promote human coronavirus infection
Source: Genome Med. 2022 Jan 27;14:10. doi: 10.1186/s13073-022-01013-1 (PMC8792531; doi:10.1186/s13073-022-01013-1)
Supplement: Supplementary file 1 — Additional file 1. Supplementary Methods, Figures, and Tables. [file 13073_2022_1013_MOESM1_ESM.docx]

**Additional File 1: Supplementary Methods, Tables, and Figures**

**Generation of the Vervet-specific genome-wide sgRNA library**

This new CRISPR knockout library was generated as previously described *(1)*. In detail, genome assembly and annotations for *Chlorocebus sabaeus* version 1.1 release 99 were downloaded from ENSEMBL and CDS-exon sequences were extracted on a per-gene basis *(2)*.  FlashFry 1.7.5 *(3)* was run for the extracted sequences with arguments "--maxMismatch 5 --maximumOffTargets 1500 --positionOutput=true" for Cas9 on-target guide identification along with the identification of potential off-targets on the entire genome. Identified guides with more than 1500 predicted off-targets or with off-targets targeting other genes without mismatches were directly discarded. As an exception to this rule, off-targets without mismatches were allowed on gene paralogs (determined by gene name similarity) in order to still allow for the generation of guides for genes with highly similar paralogs. Remaining guides were ranked by the formula below and the best four guides for each gene were selected.

score = bonus_domain_ + bonus_early_exon_ + 0.7 * score_azimuth_ + 0.3 * score_cfd_

where bonus_domain_ was 0.14 if the guide would cut in an annotated protein domain, as determined by Pfam 31 (cite:  doi: 10.1093/nar/gky995), otherwise 0; bonus_early_exon_ was 0.08 if the cut site was in the first half of the gene's exons; score_azimuth_ reflects the on-target efficacy and score_cfd_ the risk of off-targets *(1)*. Overall, using the PAVOOC sgRNA designer tool, 75,433 sgRNAs were designed to target protein functional domains of 18,873 Vervet genes (*Chlorocebus sabaeus*) *(4)*.

The sgRNAs are presented in Additional file 10: Table S9.

The sgRNA pool was synthesized as an oligo pool (GenScript). To generate double-strand DNA linkers compatible for cloning in the BsmBI sites of the LentiCRISPRv2 vector (Addgene 52961), 13 PCR reactions (final volume 25 µl) were carried out using 1 ng DNA template per reaction, 0.5 µM of each Pool-Array-For (5’ taacttgaaagtatttcgatttcttggctttatatatcttgtggaaaggacgaaacaccg 3’) and Pool-Array-Rev (5’ actttttcaagttgataacggactagccttattttaacttgctatttctagctctaaaac 3’) primers, in 1X NEBNext High Fidelity PCR Master Mix (New England BioLabs). The PCR program was as follows: initial denaturation at 98°C for 30 s, 17 cycles of denaturation at 98°C for 10 s, annealing at 63°C for 10 s, extension at 72°C for 15 s, and final extension at 72°C for 2min. The 13 PCR reactions were pooled and gel-purified on a 2% agarose gel using the PureLink Quick Gel Extraction Kit (Thermo Fisher Scientific). The oligo-pool amplicon was cloned in the LentiCRISPRv2 plasmid, previously dephosphorylated and digested with Esp3I using the Gibson Assembly Master Mix (New England BioLabs). A total of 10 Gibson reactions were carried out using a ratio of 50 ng amplicon for 330 ng plasmid. An additional Gibson reaction was carried without amplicon to assess the ligation efficiency. The Gibson reactions were pooled, isopropanol precipitated followed by a 70% ethanol wash and the pellet was resuspended in 30 µl of water. To amplify the library, 2 µl of the concentrated ligation mix were transformed into 25 µl of Endura ElectroCompetent cells (Lucigen) in 0.1 cm gap cuvettes (Bio-Rad) for a total of 8 electroporations, using the Gene Pulser Xcell electroporator (Bio-Rad) and the following settings: 10 μF Capacitance, 600 Ohms Resistance and 1800 V Voltage. The no-insert ligation was transformed as for the plus-amplicon Gibson assembly reactions. The transformed bacteria were plated on 14 large 150 mm X 10 mm petri dishes. The coverage was calculated using a 1/10000 dilution plate. The layer of grown ampicillin resistant bacteria was collected, and plasmid DNA was extracted in 8 maxi preparations using the ZymoPURE II Plasmid Maxiprep Kit (Zymo Research).

**Validation of the Vervet-specific genome-wide sgRNA library**

In order to validate the new Vervet sgRNA library, it was cloned in LentiCRISPRv2 as well as the oligo pool and indexed for Illumina sequencing by PCR. 17 cycles of PCR (two reactions per sample type) were carried out as above in DNA amplification and sequencing but respectively with 50 ng and 10 ng of cloned and pre-cloned oligo pool templates. The primers are listed in Table S1. The amplicons were separated on a 2% agarose gel and gel extracted using the PureLink Quick Gel Extraction Kit (Thermo Fisher Scientific). Amplicons were sequenced using a MiSeq sequencer with the Reagent Micro Kit (2X 150 cycles) (Illumina) at the Interdisciplinary Center for Biotechnology Research (ICBR; University of Florida, Gainesville). The coverage of the Vervet library (i.e. number of times each sgRNA is represented) was estimated to be 800X based on the number of colonies on the 1/10000 bacteria dilution plate. The normalized sgRNA counts were generated using MAGeCK *(5)*. Both cloned and pre-cloned distributions met the quality criteria **(Fig. S2A-2B)**. Indeed, the Skew values calculated from the sgRNA frequencies, respectively 1.81 and 2.25 for the cloned and oligo-pool, are considered good when they are less than 10 and close to 1 *(6, 7)*. The 90^th^ percentile to 10^th^ percentile ratio, respectively 5.5 and 4.5 for the cloned and oligo-pool were less than 10-fold, indicating a uniform distribution*(7)*. Similarly, the Gini coefficient calculated using MAGeCK was near zero, respectively 0.085 and 0.083 for the cloned and oligo-pool normalized counts, which indicates that the sgRNAs were evenly distributed. There was also a good correlation (Pearson R = 0.763) between the sgRNA count distributions before and after cloning **(Fig. S2C)**, showing that the cloning did not skew the representation.

**Genomic DNA extraction**

Genomic DNA was extracted using the Quick-DNA Midiprep Plus Kit (Zymo Research) or the DNA STAT-60 buffer (Tel-Test, Inc.) depending on product availability and following the manufacturers’ instructions. The extraction was carried out using 5 volumes of DNA STAT-60 for 1 volume of cell suspension in DNA/RNA Shield. The samples were homogenized by pipetting and DNA was precipitated by the addition of half the volume (i.e., homogenate) of ethanol. The DNA was spooled, briefly air dried, resuspended in 8 mM NaOH, and extracted with 1 volume of chloroform. The DNA in the aqueous phase was precipitated with one volume of isopropanol, pooled and washed in 1 ml of 70% ethanol. In the case of the control samples not stored in DNA/RNA Shield, the cell pellets were homogenized by adding 1 ml of DNA STAT-60 per 10 million cells.

**Table S1. DNA extraction methods**

| **Screen** | **DNA extraction method** |
| --- | --- |
| Vero E6, Vervet library:  SARS-CoV-2 samples | DNA STAT-60 |
| Vero E6, Vervet library:  OC43 samples | Quick-DNA Midiprep Plus Kit |
| HEK293T-hACE2, Brunello library Screen 1:  SARS-CoV-2 and OC43 samples | Quick-DNA Midiprep Plus Kit |
| HEK293T-hACE2, Brunello library Screen 2:  SARS-CoV-2 and OC43 samples | DNA STAT-60 |

**Comparison of our HCoV screens to previously published screens**

For our own screens, data was obtained from the sequencing service over the Illumina basespace website as fastq files. For previously published screens, we obtained two types of data. First, we obtained published top hits from “The COVID-19 Drug and Gene Set Library” (<https://maayanlab.cloud/covid19/>)  *(8)*. To allow for a fair comparison with the same data analysis standards, we also downloaded the raw data for 5 published screens and analysed them in parallel with our own screening data. For Wei et al., raw sequencing files were not available, so we used read counts as provided in their Supplemental Materials. For all others, we were able to download and process fastq files from GEO, ArrayExpress or NCBI : Schneider et al.: GSE162038; Hoffmann et al.: GSE162039; Daniloski et al.: GSE158298; Wang et al.: E-MTAB-9638; Baggen et al: PRJNA685335. CRISPR screen library guide annotations were provided by each publication or downloaded from Addgene. They are delivered with the source code (see below). The Cas9 libraries for both Vero E6 data sets (ours and Wei et al.) had been designed such that some of the sgRNAs were able to target more than one gene (this was mostly the case for highly homologous genes). In order to avoid confusion in the interpretation of the analysed data, we merged their target gene names with an underscore. Further, the two Vervet libraries were designed based on different annotations leading to incompatible annotation of unnamed genes. To improve comparability of our data set with Wei et al., we used BioPython (1.78) to map the sgRNAs of their library to the Vervet genome and extracted gene IDs for our annotation. Further, based on the sgRNA sequences, we identified the ENSEMBL IDs for ~1000 genes that had generic LOC-gene-ids and replaced them. For data from external screens, fastq-files from the same sample but different sequencing lanes were merged into one file prior to analysis. Further, only samples for OC43 and SARS-CoV-2 infections and controls were kept. Samples for infections with other viruses were discarded, i.e. not used in downstream analysis. Independent of whether single-end or paired-end sequencing was performed, only read 1 (R1) was used and read 2 was discarded.   Data analysis was facilitated by the MAGeCK-VISPR pipeline *(9)*, version 0.5.6. MAGeCK-VISPR includes a set of different tools for quality-control of the sequencing files for counting sgRNA and estimating positive- and negative-selected genes. For the latter, the pipeline relies on MAGeCK, a commonly employed tool for the analysis of CRISPR screens *(10)*, version 0.5.9.4. The tool implements the MLE- and the RRA-algorithm to identify top positive- and/or negative-selection genes, based on the enrichment of CRISPR sgRNAs in individual samples. In our data, examination of the top hits of MLE- and RRA-based analysis indicated that RRA was more reliable in identifying positive- and negative-selected genes. We thus analysed all data sets using the RRA algorithm.  Processed data were analysed and compared either manually in VISPR or by using custom-made python scripts (employed packages: python (3.8.5), numpy (1.19.1), pandas (1.1.1), matplotlib (3.3.1), matplotlib_venn_ (0.11.5), seaborn (0.11.1)). All source code for processing and analysis of the data is available on <https://github.com/moritzschaefer/covid19-screens> *(11)*.


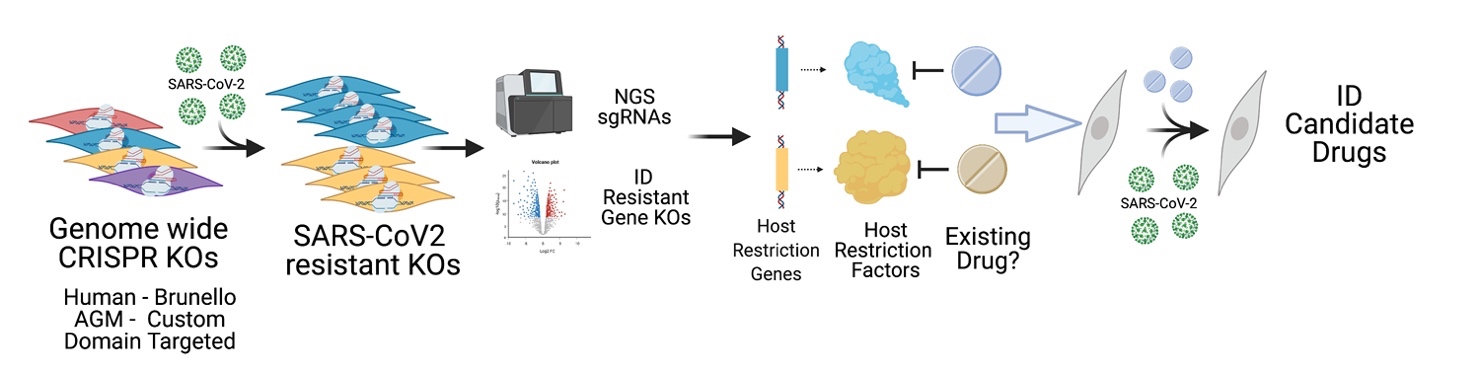


**Fig. S1. Overall study design.** The human Brunello knockout libraries were produced in the human kidney cell line HEK293T-hACE2 and the newly generated Vervet knockout libraries were made in the African green monkey epithelial cell line Vero E6. The libraries were infected with SARS-CoV-2 or OC43. The sgRNAs in cells that were resistant to viral infection were amplified and sequenced to identify candidate host factors promoting HCoV infection. Commercially available drugs targeting a subset of these host factors were tested for antiviral activity *in vitro*.

**A**


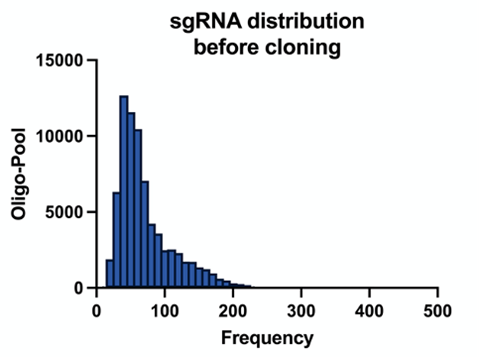


**B**


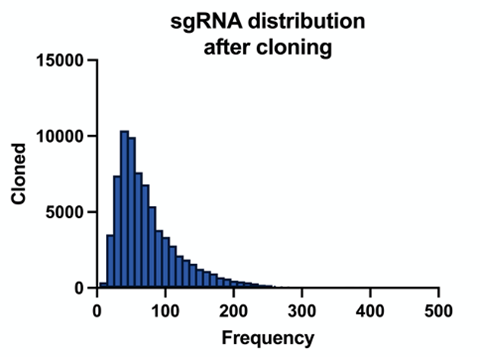


**C**


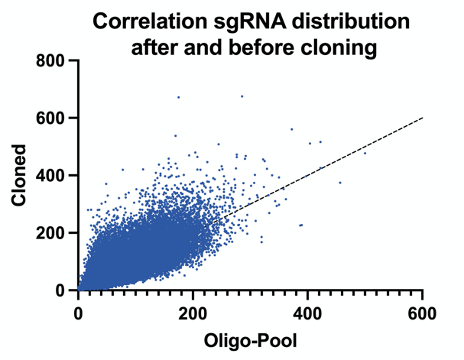


**Figure S2. Validation of the Vervet sgRNA library.**

The sgRNA distribution (frequency) of the Vervet library before and after cloning in the LentiCRISPRv2 plasmid are shown as histograms. The Correlation plot shows that the sgRNA distribution was well conserved after cloning (Pearson R=0.763).

**
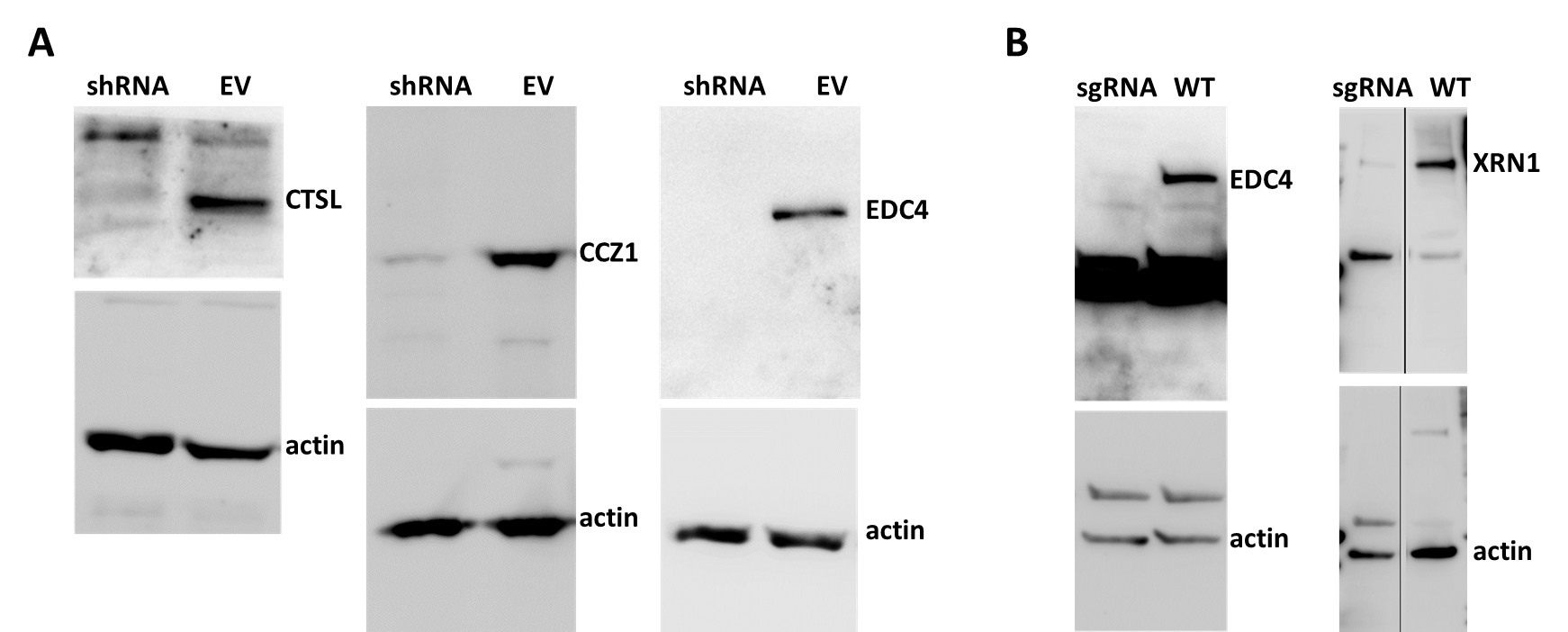
**

**Figure S3. Full-length westerns blots corresponding to cropped images in Figure 6 within the main text.** Blots in panel A correspond to Fig. 6A and blots in panel B correspond to Fig. 6C.

**Table S2: Primers used for the generation of the sgRNA amplicon libraries for Illumina Next Generation Sequencing**

| **Screen** | **Primers** | **Sequence (5'-3')** | **Barcode** | **Sample description** |
| --- | --- | --- | --- | --- |
|  | 1step-For | aatgatacggcgaccaccgagatctacactctttccctacacgacgctcttccgatctnnnnngctttatatatcttgtggaaaggacgaaacacc | N/A | Brunello and Vervet samples |
| **CRISPR Knockout Screen I Brunello library Hek293T-ACE2** | 1step-R01 | caagcagaagacggcatacgagataagtagaggtgactggagttcagacgtgtgctcttccgatctccgactcggtgccactttttcaa | aagtagag | T0puro 1 |
|  | 1step-R02 | caagcagaagacggcatacgagatacacgatcgtgactggagttcagacgtgtgctcttccgatctccgactcggtgccactttttcaa | acacgatc | T0puro 2 |
|  | 1step-R03 | caagcagaagacggcatacgagatcgcgcggtgtgactggagttcagacgtgtgctcttccgatctccgactcggtgccactttttcaa | cgcgcggt | T0puro 3 |
|  | 1step-R04 | caagcagaagacggcatacgagatcatgatcggtgactggagttcagacgtgtgctcttccgatctccgactcggtgccactttttcaa | catgatcg | T0puro 4 |
|  | 1step-R06 | caagcagaagacggcatacgagattccttggtgtgactggagttcagacgtgtgctcttccgatctccgactcggtgccactttttcaa | tccttggt | Sars-Cov2 First infection (0.01) 1 |
|  | 1step-R07 | caagcagaagacggcatacgagataacgcattgtgactggagttcagacgtgtgctcttccgatctccgactcggtgccactttttcaa | aacgcatt | Sars-Cov2 First infection (0.01) 2 |
|  | 1step-R08 | caagcagaagacggcatacgagatacaggtatgtgactggagttcagacgtgtgctcttccgatctccgactcggtgccactttttcaa | acaggtat | Sars-Cov2 First infection (0.01) 3 |
|  | 1step-R09 | caagcagaagacggcatacgagataggtaagggtgactggagttcagacgtgtgctcttccgatctccgactcggtgccactttttcaa | aggtaagg | Sars-Cov2 First infection (0.01) 4 |
|  | 1step-R10 | caagcagaagacggcatacgagatactgtatcgtgactggagttcagacgtgtgctcttccgatctccgactcggtgccactttttcaa | actgtatc | Sars-Cov2 Reinfection (0.1) 1 |
|  | 1step-R11 | caagcagaagacggcatacgagataggtcgcagtgactggagttcagacgtgtgctcttccgatctccgactcggtgccactttttcaa | aggtcgca | Sars-Cov2 Reinfection (0.1) 2 |
|  | 1step-R12 | caagcagaagacggcatacgagatataggtccgtgactggagttcagacgtgtgctcttccgatctccgactcggtgccactttttcaa | ataggtcc | Sars-Cov2 Reinfection (0.1) 3 |
|  | 1step-R13 | caagcagaagacggcatacgagatcagtgcttgtgactggagttcagacgtgtgctcttccgatctccgactcggtgccactttttcaa | cagtgctt | Sars-Cov2 Reinfection (0.01) 1 |
|  | 1step-R14 | caagcagaagacggcatacgagataccatagggtgactggagttcagacgtgtgctcttccgatctccgactcggtgccactttttcaa | accatagg | Sars-Cov2 Reinfection (0.01) 2 |
|  | 1step-R15 | caagcagaagacggcatacgagatcaacttgggtgactggagttcagacgtgtgctcttccgatctccgactcggtgccactttttcaa | caacttgg | Sars-Cov2 Reinfection (0.01) 3 |
|  | 1step-R16 | caagcagaagacggcatacgagatgacgaactgtgactggagttcagacgtgtgctcttccgatctccgactcggtgccactttttcaa | gacgaact | Sars-Cov2 Prolonged (0.1) 1 |
|  | 1step-R20 | caagcagaagacggcatacgagatagttcgcagtgactggagttcagacgtgtgctcttccgatctccgactcggtgccactttttcaa | agttcgca | Sars-Cov2 Prolonged (0.1) 2 |
|  | 1step-R21 | caagcagaagacggcatacgagatctgtatgcgtgactggagttcagacgtgtgctcttccgatctccgactcggtgccactttttcaa | ctgtatgc | Sars-Cov2 Prolonged (0.1) 3 |
|  | 1step-R22 | caagcagaagacggcatacgagattctaggaggtgactggagttcagacgtgtgctcttccgatctccgactcggtgccactttttcaa | tctaggag | Sars-Cov2 Prolonged (0.01) 1 |
|  | 1step-R23 | caagcagaagacggcatacgagattgcttgctgtgactggagttcagacgtgtgctcttccgatctccgactcggtgccactttttcaa | tgcttgct | Sars-Cov2 Prolonged (0.01) 2 |
|  | 1step-R24 | caagcagaagacggcatacgagatacgaacgagtgactggagttcagacgtgtgctcttccgatctccgactcggtgccactttttcaa | acgaacga | Sars-Cov2 Prolonged (0.01) 3 |
|  | 1step-R25 | caagcagaagacggcatacgagatttcgccatgtgactggagttcagacgtgtgctcttccgatctccgactcggtgccactttttcaa | ttcgccat | OC43 First infection (0.1) 1 |
|  | 1step-R26 | caagcagaagacggcatacgagatgagcaatcgtgactggagttcagacgtgtgctcttccgatctccgactcggtgccactttttcaa | gagcaatc | OC43 First infection (0.1) 2 |
|  | 1step-R27 | caagcagaagacggcatacgagataacactgggtgactggagttcagacgtgtgctcttccgatctccgactcggtgccactttttcaa | aacactgg | OC43 First infection (0.1) 3 |
|  | 1step-R28 | caagcagaagacggcatacgagatccatgaacgtgactggagttcagacgtgtgctcttccgatctccgactcggtgccactttttcaa | ccatgaac | OC43 First infection (0.1) 4 |
|  | 1step-R29 | caagcagaagacggcatacgagatatagtcgggtgactggagttcagacgtgtgctcttccgatctccgactcggtgccactttttcaa | atagtcgg | OC43 First infection (0.1) 5 |
|  | 1step-R30 | caagcagaagacggcatacgagatgattgtccgtgactggagttcagacgtgtgctcttccgatctccgactcggtgccactttttcaa | gattgtcc | OC43 Reinfection (0.1) 1 |
|  | 1step-R31 | caagcagaagacggcatacgagattccacgttgtgactggagttcagacgtgtgctcttccgatctccgactcggtgccactttttcaa | tccacgtt | OC43 Reinfection (0.1) 2 |
|  | 1step-R32 | caagcagaagacggcatacgagatcaactccagtgactggagttcagacgtgtgctcttccgatctccgactcggtgccactttttcaa | caactcca | OC43 Reinfection (0.1) 5 |
|  | 1step-R36 | caagcagaagacggcatacgagataagtcctcgtgactggagttcagacgtgtgctcttccgatctccgactcggtgccactttttcaa | aagtcctc | OC43 Reinfection (0.01) 1 |
|  | 1step-R37 | caagcagaagacggcatacgagatgttcttcggtgactggagttcagacgtgtgctcttccgatctccgactcggtgccactttttcaa | gttcttcg | OC43 Reinfection (0.01) 2 |
|  | 1step-R38 | caagcagaagacggcatacgagatacagtgacgtgactggagttcagacgtgtgctcttccgatctccgactcggtgccactttttcaa | acagtgac | OC43 Reinfection (0.01) 5 |
| **CRISPR Knockout Screen II Brunello library Hek293T-ACE2** | **1step-R01** | caagcagaagacggcatacgagataagtagaggtgactggagttcagacgtgtgctcttccgatctccgactcggtgccactttttcaa | aagtagag | T0puro 1 |
|  | 1step-R02 | caagcagaagacggcatacgagatacacgatcgtgactggagttcagacgtgtgctcttccgatctccgactcggtgccactttttcaa | acacgatc | T0puro 2 |
|  | 1step-R03 | caagcagaagacggcatacgagatcgcgcggtgtgactggagttcagacgtgtgctcttccgatctccgactcggtgccactttttcaa | cgcgcggt | T0puro 3 |
|  | 1step-R10 | caagcagaagacggcatacgagataacaatgggtgactggagttcagacgtgtgctcttccgatctccgactcggtgccactttttcaa | aacaatgg | Sars-Cov2 First infection (0.3) 1 |
|  | 1step-R11 | caagcagaagacggcatacgagatactgtatcgtgactggagttcagacgtgtgctcttccgatctccgactcggtgccactttttcaa | actgtatc | Sars-Cov2 First infection (0.3) 2 |
|  | 1step-R12 | caagcagaagacggcatacgagataggtcgcagtgactggagttcagacgtgtgctcttccgatctccgactcggtgccactttttcaa | aggtcgca | Sars-Cov2 First infection (0.3) 3 |
|  | 1step-R13 | caagcagaagacggcatacgagatataggtccgtgactggagttcagacgtgtgctcttccgatctccgactcggtgccactttttcaa | ataggtcc | Sars-Cov2 Reinfection (0.03) 1 |
|  | 1step-R14 | caagcagaagacggcatacgagatcagtgcttgtgactggagttcagacgtgtgctcttccgatctccgactcggtgccactttttcaa | cagtgctt | Sars-Cov2 Reinfection (0.03) 2 |
|  | 1step-R20 | caagcagaagacggcatacgagatagttcgcagtgactggagttcagacgtgtgctcttccgatctccgactcggtgccactttttcaa | agttcgca | Sars-Cov2 Reinfection (0.03) 3 |

| **CRISPR Knockout Screen Vervet library Vero-E6** | 1step-R01 | caagcagaagacggcatacgagataagtagaggtgactggagttcagacgtgtgctcttccgatctccgactcggtgccactttttcaa | aagtagag | T0puro 1 |
| --- | --- | --- | --- | --- |
|  | 1step-R02 | caagcagaagacggcatacgagatacacgatcgtgactggagttcagacgtgtgctcttccgatctccgactcggtgccactttttcaa | acacgatc | T0puro 2 |
|  | 1step-R03 | caagcagaagacggcatacgagatcgcgcggtgtgactggagttcagacgtgtgctcttccgatctccgactcggtgccactttttcaa | cgcgcggt | T0puro 3 |
|  | 1step-R04 | caagcagaagacggcatacgagatcatgatcggtgactggagttcagacgtgtgctcttccgatctccgactcggtgccactttttcaa | catgatcg | Sars-Cov2 Reinfection (0.1) 1 |
|  | 1step-R05 | caagcagaagacggcatacgagatcgttaccagtgactggagttcagacgtgtgctcttccgatctccgactcggtgccactttttcaa | cgttacca | Sars-Cov2 Reinfection (0.1) 2 |
|  | 1step-R06 | caagcagaagacggcatacgagattccttggtgtgactggagttcagacgtgtgctcttccgatctccgactcggtgccactttttcaa | tccttggt | Sars-Cov2 Reinfection (0.1) 3 |
|  | 1step-R07 | caagcagaagacggcatacgagataacgcattgtgactggagttcagacgtgtgctcttccgatctccgactcggtgccactttttcaa | aacgcatt | Sars-Cov2 Reinfection (0.1) 4 |
|  | 1step-R08 | caagcagaagacggcatacgagatacaggtatgtgactggagttcagacgtgtgctcttccgatctccgactcggtgccactttttcaa | acaggtat | OC43 First infection (0.1) 1 |
|  | 1step-R09 | caagcagaagacggcatacgagataggtaagggtgactggagttcagacgtgtgctcttccgatctccgactcggtgccactttttcaa | aggtaagg | OC43 First infection (0.01) 2 |
|  | 1step-R10 | caagcagaagacggcatacgagataacaatgggtgactggagttcagacgtgtgctcttccgatctccgactcggtgccactttttcaa | aacaatgg | OC43 First infection (0.01) 3 |
|  | 1step-R11 | caagcagaagacggcatacgagatactgtatcgtgactggagttcagacgtgtgctcttccgatctccgactcggtgccactttttcaa | actgtatc | OC43 First infection (0.01) 4 |
|  | 1step-R12 | caagcagaagacggcatacgagataggtcgcagtgactggagttcagacgtgtgctcttccgatctccgactcggtgccactttttcaa | aggtcgca | OC43 Reinfection (0.1) 1 |
|  | 1step-R13 | caagcagaagacggcatacgagatataggtccgtgactggagttcagacgtgtgctcttccgatctccgactcggtgccactttttcaa | ataggtcc | OC43 Reinfection (0.1) 2 |
|  | 1step-R14 | caagcagaagacggcatacgagatcagtgcttgtgactggagttcagacgtgtgctcttccgatctccgactcggtgccactttttcaa | cagtgctt | OC43 Reinfection (0.1) 3 |
|  | 1step-R15 | caagcagaagacggcatacgagataccatagggtgactggagttcagacgtgtgctcttccgatctccgactcggtgccactttttcaa | accatagg | OC43 Reinfection (0.1) 4 |

| **Vervet library validation** | Pool-R48 | caagcagaagacggcatacgagattactagcggtgactggagttcagacgtgtgctcttccgatctcttattttaacttgctatttctagctctaaaac | tactagcg | Vervet-PAVOOC Oligo-pool |
| --- | --- | --- | --- | --- |
|  | 1step-R34 | caagcagaagacggcatacgagattgagctgtgtgactggagttcagacgtgtgctcttccgatctccgactcggtgccactttttcaa | tgagctgt | Vervet-PAVOOC Cloned library |

In the Sample description column, the MOI employed in this study are shown between brackets and the replicate samples (three to five) per condition are indicated by their number (end of the description). For the Vervet screen, we did not collect and sequence the Sars-Cov2 first infection (0.01) samples.

**SUPPLEMENTARY REFERENCES**

1. J. G. Doench, N. Fusi, M. Sullender, M. Hegde, E. W. Vaimberg, K. F. Donovan, I. Smith, Z. Tothova, C. Wilen, R. Orchard, H. W. Virgin, J. Listgarten, D. E. Root, Optimized sgRNA design to maximize activity and minimize off-target effects of CRISPR-Cas9. *Nature Biotechnology* **34**, 184–191 (2016).

2. W. C. Warren, A. J. Jasinska, R. García-Pérez, H. Svardal, C. Tomlinson, M. Rocchi, N. Archidiacono, O. Capozzi, P. Minx, M. J. Montague, K. Kyung, L. W. Hillier, M. Kremitzki, T. Graves, C. Chiang, J. Hughes, N. Tran, Y. Huang, V. Ramensky, O.-W. Choi, Y. J. Jung, C. A. Schmitt, N. Juretic, J. Wasserscheid, T. R. Turner, R. W. Wiseman, J. J. Tuscher, J. A. Karl, J. E. Schmitz, R. Zahn, D. H. O’Connor, E. Redmond, A. Nisbett, B. Jacquelin, M. C. Müller-Trutwin, J. M. Brenchley, M. Dione, M. Antonio, G. P. Schroth, J. R. Kaplan, M. J. Jorgensen, G. W. C. Thomas, M. W. Hahn, B. J. Raney, B. Aken, R. Nag, J. Schmitz, G. Churakov, A. Noll, R. Stanyon, D. Webb, F. Thibaud-Nissen, M. Nordborg, T. Marques-Bonet, K. Dewar, G. M. Weinstock, R. K. Wilson, N. B. Freimer, The genome of the vervet (Chlorocebus aethiops sabaeus). *Genome Res* **25**, 1921–1933 (2015).

3. A. McKenna, J. Shendure, FlashFry: a fast and flexible tool for large-scale CRISPR target design. *BMC Biol* **16**, 74 (2018).

4. M. Schaefer, D.-A. Clevert, B. Weiss, A. Steffen, PAVOOC: designing CRISPR sgRNAs using 3D protein structures and functional domain annotations. *Bioinformatics* **35**, 2309–2310 (2019).

5. W. Li, H. Xu, T. Xiao, L. Cong, M. I. Love, F. Zhang, R. A. Irizarry, J. S. Liu, M. Brown, X. S. Liu, MAGeCK enables robust identification of essential genes from genome-scale CRISPR/Cas9 knockout screens. *Genome Biol* **15**, 554 (2014).

6. M. Wegner, K. Husnjak, M. Kaulich, Unbiased and Tailored CRISPR/Cas gRNA Libraries by Synthesizing Covalently-closed-circular (3Cs) DNA. *Bio-protocol* **10**, e3472 (2020).

7. J. Joung, S. Konermann, J. S. Gootenberg, O. O. Abudayyeh, R. J. Platt, M. D. Brigham, N. E. Sanjana, F. Zhang, Genome-scale CRISPR-Cas9 knockout and transcriptional activation screening. *Nat Protoc* **12**, 828–863 (2017).

8. The COVID-19 Drug and Gene Set Library (available at https://maayanlab.cloud/covid19).

9. W. Li, J. Köster, H. Xu, C.-H. Chen, T. Xiao, J. S. Liu, M. Brown, X. S. Liu, Quality control, modeling, and visualization of CRISPR screens with MAGeCK-VISPR. *Genome Biology* **16**, 281 (2015).

10. W. Li, H. Xu, T. Xiao, L. Cong, M. I. Love, F. Zhang, R. A. Irizarry, J. S. Liu, M. Brown, X. S. Liu, MAGeCK enables robust identification of essential genes from genome-scale CRISPR/Cas9 knockout screens. *Genome Biology* **15**, 554 (2014).

11. M. Schäefer, covid19-screens*GitHub* (available at https://github.com/moritzschaefer/covid19-screens).
